# Relationship between neutrophil–lymphocyte ratio and short-term prognosis in the chronic obstructive pulmonary patients with acute exacerbation

**DOI:** 10.1042/BSR20190675

**Published:** 2019-05-14

**Authors:** Jia Liu, Jie Liu, Yong Zou

**Affiliations:** 1 Department of Emergency Medicine, Xiangya Hospital, Central South University, Changsha, Hunan 410008, China; 2 Department of Emergency Medicine, The University of Hong Kong-Shenzhen Hospital, Shenzhen, Guangdong 518053, China

**Keywords:** chronic obstructive pulmonary patients, COPD, neutrophil-lymphocyte ratio, prognosis

## Abstract

We retrospectively collected data from a large sample size of population and explore the relationship between neutrophil–lymphocyte ratio (NLR) and adverse outcomes, and assessed the clinical prognostic utility of NLR in patients with chronic obstructive pulmonary patients with acute exacerbation (AECOPD). We reviewed 3 years medical case records, 622 patients were identified including 48 died and 574 alive. Compared with alive group, the died group had significantly elevated neutrophils, lymphocyte, and NLR level (*P*<0.001). The high-sensitive C-protein level of died group was also higher compared with alive group (7.48 ± 4.2 vs 1.26 ± 0.56, vs *P*<0.001). The univariate logistic regression indicated that elevated NLR level was associated with the increased of adverse outcome (odds ratio [OR] = 4.59, 95% CI: 2.27–8.94, *P*<0.001). After adjusted potential confounding factors, the elevated NLR level was still associated with adverse outcomes in the chronic obstructive pulmonary patients with acute exacerbation (OR = 2.05, 95% CI: 1.21–3.48, *P*=0.008). The area under the receiver operating characteristic curve for death at 90 days was 0.742 (95% CI: 0.554–0.881). NLR cutoff of >4.19 had a sensitivity of 71.4% and specificity of 74.2%. Our results suggested that NLR, as a rapid, inexpensive and easily obtained blood routine index was associated with short-term adverse outcomes in patients with AECOPD. The elevated NLR predicted the increased the risk of 90-day mortality in patients with AECOPD.

## Introduction

Chronic obstructive pulmonary disease (COPD) is a type of obstructive lung disease characterized by long-term breathing problems and poor airflow. The main symptoms include shortness of breath and cough with sputum production. COPD is a progressive disease, meaning it typically worsens over time. Eventually everyday activities, such as walking or getting dressed, become difficult. COPD, contributing significantly to chronic morbidity and mortality, is responsible for around 6% of all deaths worldwide in 2012, and will be the fifth public health burden and third leading cause of death by 2020. COPD has been an important public health problem in the world [1,2]. In China, the prevalence rate is high and up to 13–30% over 60 years old. With the advent of the aging society, the incidence will continue to rise [3]. Acute exacerbations of COPD (AECOPD) are part of the natural history of this disease where a large proportion of mortality and health expenditure is encountered [4]. It is estimated that approximately 4% of the general populations in the western world is admitted with an AECOPD as least once a year and nearly 1/5 of hospital visits is due to AECOPD [5]. The reasons of exacerbations still remain unclear, but it is suggested that 70% of AECOPD is associated with be infectious triggered by origin viruses [6]. Previous studies have suggested that inflammation is one of the pathologic triads of COPD (others included protease–antiprotease imbalance, and oxidative stress), and bronchitis and emphysema are the two main clinical phenotypes of the disease [7–9]. Inflammation in COPD is amplified during exacerbation episodes in comparison with stable periods and increased levels of inflammatory markers that are associated with lung function decline [10,11]. The presence of systemic inflammation strongly affects quality of life and increases mortality, leads to weight loss, muscle wasting, and tissue depletion in COPD patients [12].

As we all know, forced expiratory volume in 1s (FEV1) is the most widely used marker to assess COPD severity. However, this index is poorly correlated with some symptoms and may not reflect the inflammatory status. Moreover, this FEV1 is not routinely used during the acute exacerbation [13,14]. Therefore, others biomarkers are needed to aid with diagnosis and treatment. The neutrophil–lymphocyte ratio (NLR) is a quick, easy, and inexpensive index, which can be easily obtained from blood routine examination. Recent studies have reported that NLR could be a new inflammatory marker to assess the inflammation in many diseases, including inflammatory bowel diseases [15], pancreatitis [16], coronary syndrome, and even tumor [17]. It is also reported that NLR can be an independent predictor in appendicitis [18]. Considering the severity of AECOPD and the close relationship between NLR and inflammation, we retrospectively collected data from a large sample size of population and explore the relationship between NLR and adverse outcomes and assessed the clinical prognostic utility of NLR in patients with chronic obstructive pulmonary patients with acute exacerbation.

## Materials and method

### Study population

We retrospectively and consecutively enrolled AECOPD-related admissions to Department of Emergency, Xiangya Hospital Central South University during the calendar months of January 2016 to December 2018. We collected follow-up information of these patients during the stable period 3 months after an acute exacerbation. Criteria for inclusion: patients with COPD was diagnosed by a pulmonary specialist based on past smoking history, clinical evaluation, and pulmonary function tests showing airflow obstruction according to the golden criteria (FEV1 <80% predicted, FEV1/FVC <70%, and bronchodilatation effect <12%) [19]; patients were primary diagnosis of AECOPD and the age of patients were more than 40 years old. AECOPD was defined as a history of increased breathlessness and at least two of the following symptoms for 24 h or more increased cough frequency or severity, sputum volume or purulence and wheeze. Criteria for admission were accepted as follows old, to have frequent exacerbation, impaired activities of daily living because shortness of breath, fever and/or deterioration in mental status, despite optimal treatment increased oxygen demand (PaO_2_ < 60 mmHg, SaO_2_ < 90%) and/or hypercapnia (over than 50 mmHg) [20]. The following patients were excluded: patients with a history of antibiotic treatment, use of systemic steroids with a prednisolone equivalent to >20 mg/day in the preceding two months, with disorders such as bronchiectasis (radiologically proven or history of phlegm expectoration >30 ml/day), tuberculosis, asthma, pulmonary fibrosis or embolism, or other inflammatory diseases such as malignancy, arthritis, inflammatory bowel diseases, or connective tissue disorders, were excluded. The study was approved by the Ethics Committee of Xiangya Hospital Central South University. The research was carried out in accordance with the World Medical Association Declaration of Helsinki, and all subjects provided written informed consent.

### Data collection

Peripheral venous blood samples were obtained using ethylenediaminetetraacetic acid-containing blood collector within 24 h after the admission. Demographic information (age, gender), smoking was defined as current smoking or smoked daily previously. Alcohol assumption was defined as >2 times a month [21,22], body mass index (BMI), systolic blood pressure and diastolic blood pressure, co-morbidities, length of stay, and history of medications) and the blood count parameters, including, white blood cell, neutrophils, lymphocyte, eosinophils, red blood cell, hemoglobin, high-sensitive C-protein, and red cell distribution width, were collected from the medical record information. NLR was calculated as the ratio of neutrophils to lymphocytes, both of which were obtained from the same automated blood samples for the study. Hematologic testing was conducted on the Beckman Coulter LH-750 Hematology Analyzer (Beckman Coulter, Inc., Fullerton, CA, U.S.A.), automated hematology analyzer. The D-dimer and fibrinogen level were measured using an automated latex-enhanced immunoassay method. Serum creatinine and blood urea nitrogen (BUN) were measured on a Roche/Hitachi Modular System P (Roche Diagnostics GmbH, Mannheim, Germany) by creatinine Jaffe´, rate blanked and compensated assay. Urine concentrations of albumin were measured by immunoturbidimetric method. FEV1 was measured using a spirometer. For arterial blood gas analyses, blood was drawn from the radial artery while the patients were breathing room air. PaO_2_ and PaCO_2_, PH were analyzed with a blood gas analyzer (Radiometer ABL 800 Flex-Denmark).

### Statistical analysis

All statistical analyses were performed using SPSS 20.0. All subjects were divided into two groups (died group and alive group) according the outcomes after 3 months. The quantitative variables with normal distribution were expressed using mean ± S.D., or median with maximum and minimum. To compare means for two independent groups, the independent student’s *t* test was used, while non-parametric test was used for non-normal distribution data. The categorical data were presented using frequencies and percentages, and the Chi-square test was used for two independent categorical data. Missing baseline data were imputed with multiple imputation. Pearson correlation coefficient was calculated between NLR and C-reactive protein. To explore the relationship between NLR and outcomes of patients, the univariate and multivariate logistic regression were performed, respectively. The following parameters were included in the regression model: age, sex, smoking, drinking, number of co-morbidities, length of stay, blood pressure, blood cell count, NLR, PH, PaCO_2_, PaO_2_, FEV1, D-dimer, fibrinogen, creatinine, blood urea nitrogen, and albumin. Collinearity diagnostics within variables were applied before the regression model was built. Receiver operating characteristic curves (ROC) were constructed for the NLR and the areas under ROC curve values with 95% CI, the sensitivity and specificity were also calculated. *P*<0.05 was considered as statistical significance.

## Results

### General characteristics of all subjects

We reviewed 3 years medical case records, 652 patients were identified according to the COPD status. Total 30 patients were excluded from analysis because they were less than 40 years old (*n*=6), non-primary AECOPD (14) or other reasons for hospitalization (*n*=10). Finally, 622 AECOPD patients were included in the analysis, including 48 died and 574 alive. The general characteristic and comparison of all parameters are presented in the Table 1. After 3-month retrospective collection, the mortality for AECOPD patients after 3 months was 7.72%. The mean age of all subjects was 74.4 ± 11.3 and males accounted for 49.4%. Total 371 patients (59.6%) sated that they have history of smoking and 299 patients have history of drinking. Amongst the common co-morbid illnesses recorded were coronary artery disease (30.1%), congestive heart failure (24.0%), arrhythmia (18.5%), chronic kidneys diseases (26.4%), and osteoporosis (23.3%). The mean number of co-morbidities was 4.6. Following admission to hospital, an overwhelming majority of patients had received systemic corticosteroids, antibiotics, and bronchodilators. Significant positive correlation was observed between NLR and CRP in patients with ACECOPD (r = 0.608, *P*<0.001).

**Table 1:** Comparison of general characteristics between died group and alive gourp

Parameters	Died group (*n*=48)	Alive group (574)	χ^2^/t/u	*P*
Age, year	75.1 ± 12.2	74.3 ± 11.8	0.450	0.653
Sex male, %	26 (54.2%)	281 (48.9%)	0.481	0.488
Smoking, %	37 (77.1%)	334 (58.2%)	6.570	0.010
Drinking, %	29 (60.4%)	270 (47.0%)	3.176	0.075
BMI, kg/m^2^	26.4 ± 4.3	25.3 ± 4.1	1.779	0.076
Co-morbidity				
Coronary artery disease, %	14 (29.2%)	173 (30.1%)	0.020	0.888
Congestive heart failure, %	12 (25.0%)	137 (23.9%)	0.031	0.860
Arrhythmia, %	12 (25.0%)	103 (17.9%)	1.463	0.226
Chronic kidney disease, %	15 (31.3%)	149 (26.0%)	0.639	0.424
Osteoporosis, %	12 (25.0%)	133 (23.2%)	0.083	0.773
Number of co-morbidities, n	5.2 ± 1.7	4.5 ± 2.5	1.903	0.058
Therapy				
Antimicrobial therapy, %	48	574	0.000	1.000
Systemic corticosteroid therapy, %	47	573	0.000	1.000
Bronchodilator therapy, %	48	574	0.000	1.000
Length of stay, days	5.5 (2.2–11)	3.0 (1–6)	1.036	0.333*
Systolic blood pressure, mmHg	159.6 ± 37.5	148.5 ± 23.5	2.974	0.003
Diastolic blood pressure, mmHg	82.1 ± 23.4	80.2 ± 13.8	0.857	0.392
Respiratory frequency, n	24.6 ±12.2	22.3 ± 8.7	1.698	0.090
White blood cell, ×10^9^	8.4 ± 3.6	7.8 ± 3.1	1.271	0.204
Neutrophils, n	774.5 ± 98.2	658.9 ± 103.5	7.462	<0.001
Lymphocyte, n	143.2 ± 68.5	193.6 ± 105.1	4.663	<0.001
Neutrophils/lymphocyte ratio	7.8± 10.1	3.1 ± 6.8	4.403	<0.001
Eosinophils, × 10^9^	0.04 ± 0.1	0.19 ± 0.4	−2.589	0.010
Red blood cell, ×10^12^	5.0 ± 40.4	5.1 ± 36.8	−0.018	0.986
Hemoglobin, g/l	118.2 ± 20.0	135.7 ± 26.8	−4.421	<0.001
Red cell distribution width, %	19.2 ± 8.4	18.5 ± 9.7	0.485	0.628
High-sensitive CRP, mg/l	7.48 ± 4.2	1.26 ± 0.56	32.454	<0.001
PH < 7.3, n	19 (39.6%)	48 (8.4%)	44.923	<0.001
PaCO_2_, mmHg	63.4 ± 13.7	62.2 ± 10.6	0.735	0.463
PaO_2_, mmHg	68.5 ± 14.2	72.6 ± 18.3	−1.514	0.131
FEV1% predicted	24.6 ± 14.3	50.3 ± 16.8	−10.289	<0.001
D-dimer, mg/l	1.24 ± 3.3	0.91 ± 1.2	1.496	0.135
Fibrinogen, g/l	3.4 ± 1.3	3.9 ± 1.4	−2.389	0.017
Creatinine, umol/l	105.4 ± 65.1	98.2.1 ± 36.8	1.208	0.227
Blood urea nitrogen, mmol/l	8.2 ± 2.3	7.6 ± 4.5	0.913	0.361
Albumin, g/l	25.4 ± 9.1	33.6 ± 10.3	−2.085	0.037

* Non-parameter test

### Comparisons of parameters between died and alive group

There were no significant differences in general characteristics between died group and alive group, including age (75.1 ± 12.2 vs 74.3 ± 11.8, *P*=0.653), male ratio (54.2 vs 48.9%, *P*=0.488), alcohol assumption rate (60.4 vs 47.0%, *P*=0.075), BMI (26.4 ± 4.3 vs 25.3 ± 4.1, *P=*0.076), co-morbidity rate (all *P*>0.05). However, the smoking rate of died group was significantly higher than that in the alive group (77.1 vs 58.2%, *P=*0.010). The mean number of co-morbidities in the died group was 5.2 and it was 4.5 in the alive group. No significant difference was found. The systolic blood pressure of died group was also higher than that in the alive group (159.6 ± 37.5 vs 148.5 ± 23.5, *P=*0.003), and no significant difference was observed in diastolic blood pressure.

Compared with alive group, the died group had significantly elevated neutrophils, lymphocyte, and NLR level (*P<*0.001). The high-sensitive C-reactive protein level of died group was also higher compared with alive group (7.48 ± 4.2 vs 1.26 ± 0.56, vs *P*<0.001). The ratio of PH <3 was also higher in the died group than in the alive group (39.6 vs 8.4%, *P<*0.001). Compared with alive group, the died group has lower hemoglobin (118.2 ± 20.0 vs 135.7 ± 26.8, *P<*0.001), eosinophils (0.04 ± 0.1 vs 0.19 ± 0.4, *P=*0.010), FEV1% (24.6 ± 14.3 vs 50.3 ± 16.8, *P*<0.001), fibrinogen (3.4 ± 1.3 vs 3.9 ± 1.4, *P=*0.017) and albumin (25.4 ± 9.1 vs 33.6 ± 10.3, *P=*0.037) level. There were no significant differences in other parameters. The specific results are presented in the Table 1.

### Univariate and multivariate logistic regression

We treated the outcomes as the dependent variable, and the general parameters including, age, sex, smoking and drinking, BMI, number of co-morbidities, length of stay, systolic and diastolic blood pressure, blood cell count, lung function index, D-dimer, fibrinogen, creatinine, blood urea nitrogen, and albumin as independent variables. The univariate logistic regression (Table 2) indicated that elevated NLR level was associated with the increased of adverse outcome (OR = 4.59, 95% CI: 2.27–8.94, *P<*0.001). Smoking also increased the risk of adverse outcome (OR = 2.43, 95% CI: 1.21–4.85, *P=*0.012). Systolic blood pressure, reduced eosinophils, high-sensitive c-protein, and PH <7.3 were also related to dead outcome. After adjusted potential confounding factors, the elevated NLR level was still associated with dead outcomes in the chronic obstructive pulmonary patients with acute exacerbation (OR = 2.05, 95% CI: 1.21–3.48, *P=*0.008). Others, smoking (OR = 2.18, 95% CI: 1.05–4.52), elevated systolic blood pressure (OR = 2.17, 95% CI: 1.89–5.26), reduced hemoglobin (OR = 3.89, 95% CI: 1.98–7.66), elevated high-sensitive C-protein (OR = 4.44, 95% CI: 2.90–8.72), and PH < 7.3 (OR = 1.46, 95% CI: 1.20–2.31) were also associated with dead outcomes. The results are presented in the Table 3.

**Table 2:** Univariate logistic regression analysis of short-term prognosis for COPD with acute exacerbation

Parameter	β	SE	Waldχ^2^	*P*	OR	(95% CI)
Age, year	0.016	0.019	0.669	0.413	1.02	0.98–1.05
Sex male, %	0.207	0.169	1.654	0.291	1.23	0.68–2.23
Smoking, %	0.887	0.316	6.347	0.012	2.43	1.21–4.85
Drinking, %	0.542	0.418	0.654	0.421	1.72	0.94–3.14
BMI, kg/m^2^	0.608	0.621	1.234	0.401	1.84	0.35–2.49
Number of co-morbidities, n	−0.296	0.158	3.504	0.061	0.74	0.55–0.37
Length of stay, days	0.016	0.130	0.015	0.902	1.02	0.79–1.31
Systolic blood pressure, mmHg	1.386	0.500	7.674	0.005	4.00	1.50–10.67
Diastolic blood pressure, mmHg	0.090	0.087	1.069	0.301	1.09	0.92–1.30
Respiratory frequency, n	0.242	0.202	1.433	0.231	0.23	1.27–0.86
White blood cell, ×10^9^	0.615	0.373	2.721	0.100	1.85	0.37–2.46
Neutrophils, %	1.398	0.366	14.610	<0.001	4.05	1.98–8.29
Lymphocyte, n	1.758	0.371	22.402	<0.001	5.80	2.80–12.00
Neutrophils/lymphocyte ratio	1.523	0.389	95.593	<0.001	4.59	2.27–8.94
Eosinophils, ×10^9^	0.301	0.127	5.625	0.018	1.35	1.05–1.73
Red blood cell, ×10^12^	−0.337	0.138	15.657	0.063	0.79	0.59–1.54
Hemoglobin, g/l	−2.223	0.044	25.491	<0.001	0.80	0.73–0.87
Red cell distribution width, %	0.415	0.171	2.634	0.102	1.51	0.86–2.17
High-sensitive C-protein, mg/l	0.450	0.141	10.112	0.001	1.57	1.19–2.07
PH < 7.3, n	1.971	0.296	35.024	<0.001	7.18	3.75–13.75
PaCO_2_, mmHg	1.685	0.261	64.136	0.055	1.31	1.17–3.74
PaO_2_, mmHg	0.947	0.277	15.973	0.058	1.77	1.23–2.55
FEV1%	−0.465	0.175	17.682	0.091	0.85	0.76–1.35
D-dimer, mg/l	1.351	0.348	29.137	0.061	0.89	0.72–1.45
Fibrinogen, g/l	1.356	0.831	10.549	0.078	1.05	0.77–1.94
Creatinine, umol/l	1.483	0.151	33.473	0.073	1.04	0.82–2.97
Blood urea nitrogen, mmol/l	0.824	0.362	3.582	0.127	1.26	0.47–3.07
Albumin, g/l	0.521	0.176	1.803	0.218	3.20	0.19–4.69

**Table 3:** Multivariate logistic regression analysis of short-term prognosis for COPD with acute exacerbation

Parameter	β	SE	Waldχ^2^	*P*	OR	(95% CI)
Smoking, %	0.778	0.373	4.358	0.037	2.18	1.05–4.52
Systolic blood pressure, mmHg	0.785	0.647	16.354	0.002	2.17	1.89–5.26
Neutrophils/lymphocyte ratio	0.719	0.269	7.147	0.008	2.05	1.21–3.48
Hemoglobin, g/l	1.359	0.346	15.463	<0.001	3.89	1.98–7.66
High-sensitive CRP, mg/l	2.671	0.554	23.212	<0.001	4.44	2.90–8.72
PH < 7.3, n	0.358	1.110	4.514	0.034	1.46	1.20–2.31

### ROC analysis

**Figure 1:**
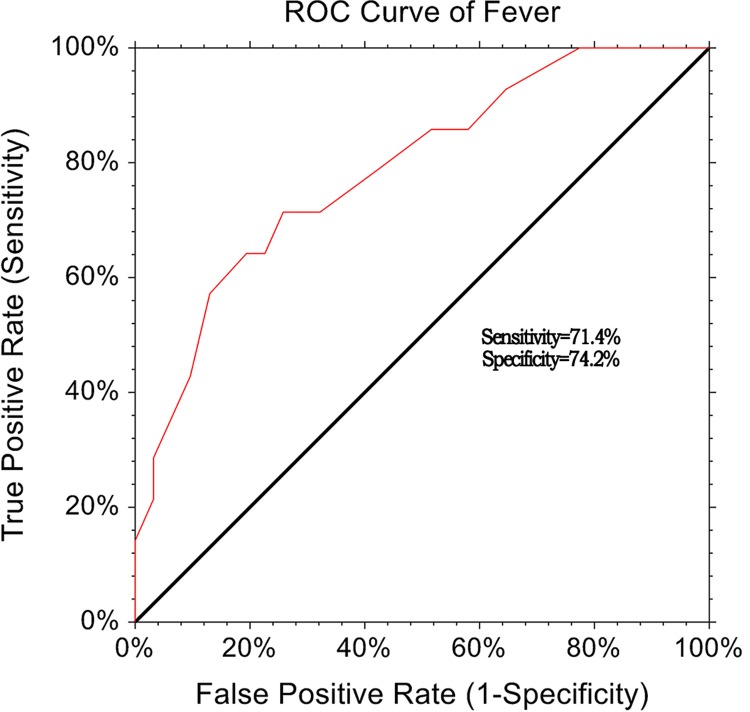
ROC curve of NLR in identifying the prognosis of AECOPD

We performed the *post hoc* to determine the prognostic utility of NLR by calculating its sensitivity and specificity using the ROC curve. The area under the ROC curve for death at 90 days and PLR was 0.742 (95% CI: 0.554–01.881). NLR cutoff of >4.19 had a sensitivity of 71.4% and specificity of 74.2% (Figure 1).

## Discussion

The present study results from multivariate indicated that NLR is associated with increased 90-day mortality in hospitalized AECOPD patients. Furthermore, NLR >4.19 provided a moderate predicted capability for short-term adverse outcome. Our results provided some supplementary information for the increasing scientific body of knowledge about the prognostic utility of NLR in not solid organ malignancies but also chronic inflammatory diseases like COPD. Previous studies also investigate the role of NLR in patients with COPD [23]. But previous study is different from the present study. The former was to identify the relationship between NLR and the severity of inflammation and recognition of acute exacerbation. The study population consisted of all types of COPD (stable and exacerbated COPD). Our study was from AECOPD patients setting. Furthermore, the present study had larger sample size (*n*=622), while the former only included 100 patients. Above all, previous study did not perform the multivariate regression analysis that made the results unstable.

It is well known that COPD is a disease characterized by airway inflammation, including stable period and acute exacerbations. The stable period was related to low level of systemic inflammation, while acute exacerbation period was usually accompanied by higher and worse inflammation status, which also increased the risk of mortality [24,25]. Therefore, early identification and management of AECOPD is of great importance in clinical practice. COPD is associated not only with an abnormal inflammatory response in the lung but also with systemic inflammation, including systemic oxidative stress, activation of circulating inflammatory cells and increased circulating levels of inflammatory cytokines [26]. Although the specific mechanism remains unclear, previous studies had suggested that some inflammatory factors, such as CRP, IL-6, and IL-8, were associated with COPD [27,28]. Elevated CRP was found in our results. As an inflammatory marker, NLR was found to be associated with prognosis in patients with AECOPD in the present study. Neutrophilic granulocyte-mediated airway inflammation is an important stage in the course of COPD. Neutrophilic granulocytes are significantly increased in the acute exacerbation phase of COPD caused by bacterial infection, but the increase does not only exist in the case of bacterial infection [29]. In COPD patients, a large number of neutrophils adhere to the airway endothelial cells and migrate to the respiratory tract under the action of interleukin 8, leukotriene B4 and other neutrophils chemotactic factors. Cytokine stimulation increases the number of neutrophils that accumulate in the respiratory tract, which in turn releases oxygen radicals and proteolytic enzymes, leading to alveolar collapse and emphysema [30]. Lung tissues of COPD patients can generate acquired immunity under the action of tobacco, bacteria, virus, and extracellular matrix lysates [31]. The main cells of action are cytotoxic CD8^+^ cells, Th1, and CD4^+^ cells. The significantly increased number of CD8^+^T lymphocytes in lung tissue can reflect the severity of airflow restriction and emphysema, and CD8^+^ cells can release perforin and granulocyte after activation, inducing apoptosis of structural cells [32]. Kim et al. proved that CD8^+^ cells play an important role in the early small airway structural remodeling in COPD patients [33]. On the other hand, lymphocytosis is correlated with age, nutritional status, and metabolic abnormalities. Studies have shown that NLR levels in patients with COPD complicated with metabolic syndrome [34]. This can partly explain the reasons. Our results also indicated that neutrophils and lymphocytes were significantly elevated. We also assessed the clinical utility of NLR for short-term prognosis. The present results indicated that the NLR has a moderate diagnostic ability for short-term prognosis in AECOPD patients. NLR cutoff of >4.19 had a sensitivity of 71.4% and specificity of 74.2%, which is similar to previous study. Raymond found that NLR could be beneficial for the early detection of potential acute exacerbations in patients with COPD who have normal levels of traditional markers. The sensitivity for detecting exacerbation of COPD was 0.768 and specificity was 0.731. The neutrophil/lymphocyte ratio is a better addition to C-reactive protein than CD64 index as a marker for infection in COPD [35]. But the present study has a small sample size (72 AECOPD patients). Previous studies also compared the clinical utility of NLR, CRP, and white blood cell and suggested that NLR is the most sensitive parameter as an indicator of exacerbated COPD. For an NLR cutoff of 3.29, sensitivity for detecting exacerbation of COPD was 80.8% and specificity was 77.7% [36]. These studies focussed on detection of AECOPD from COPD. Our results further suggested that NLR can be a useful predictive factor in patients with AECOPD. Although the diagnosis of AECOPD is usually based on clinical symptoms, an elevated NLR value that can be detected during routine checkups of stable COPD patients may lead the way in the early detection of AECOPD.

The present study has several limitations. First, our study is based on retrospective study design. There may be some selective bias. Second, we have tried our best to collect the clinical information. But there were still some clinical parameters that were not included in the analysis such as electrolyte level. Too many missing data were found for these parameters. Third, we only used one measure in time rather than serial measure. Finally, the present results were from the short-term follow-up. Confirmatory studies need to be performed, particularly in a larger cohort study.

In conclusion, the present study suggested that NLR, as a rapid, inexpensive and easily obtained blood routine index, was associated with short-term adverse outcomes in patients with AECOPD. The elevated NLR predicted the increased the risk of 90-day mortality in patients with AECOPD. NLR may be an independent predictor. Future studies should explore the specific molecular mechanism and focussed on long-term outcome. Patients may benefit from regular clinical surveillance for NLR.

## Abbreviations

AECOPD: acute exacerbation chronic obstructive pulmonary disease
BMI: body mass index
BUN: blood urea nitrogen
FEV1: forced expiratory volume in 1s
NLR: neutrophil–lymphocyte ratio
OR: odds ratio
ROC: receiver operating characteristic curve
